# Different Facets of Copy Number Changes: Permanent, Transient, and Adaptive

**DOI:** 10.1128/MCB.00652-15

**Published:** 2016-03-18

**Authors:** Sweta Mishra, Johnathan R. Whetstine

**Affiliations:** Massachusetts General Hospital Cancer Center and Department of Medicine, Harvard Medical School, Charlestown, Massachusetts, USA

## Abstract

Chromosomal copy number changes are frequently associated with harmful consequences and are thought of as an underlying mechanism for the development of diseases. However, changes in copy number are observed during development and occur during normal biological processes. In this review, we highlight the causes and consequences of copy number changes in normal physiologic processes as well as cover their associations with cancer and acquired drug resistance. We discuss the permanent and transient nature of copy number gains and relate these observations to a new mechanism driving transient site-specific copy gains (TSSGs). Finally, we discuss implications of TSSGs in generating intratumoral heterogeneity and tumor evolution and how TSSGs can influence the therapeutic response in cancer.

## INTRODUCTION

It was long thought that the DNA sequences of healthy individuals were 99.9% identical to each other (1). However, genome-wide sequencing efforts in individuals from multiple ethnicities have revealed more variations in the genetic architecture than were previously appreciated (2–4).

**FIG 1:**
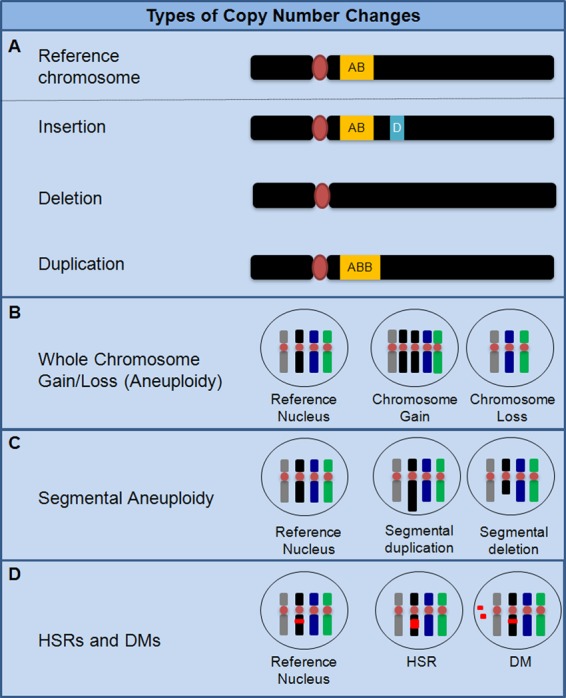
Types of copy number changes. (A) Representative examples of structural chromosomal alterations are shown, with a new sequence insertion (D), deletion of region AB, and duplication of sequence B (ABB). The reference chromosome is shown at the top. (B) Aneuploidy with whole chromosome gain (the extra black chromosome) and loss (of black chromosome) are depicted with respect to a normal mitotic reference nucleus. (C) A part of a chromosome (black) can be amplified or deleted (black), giving rise to segmental aneuploidy. This is demonstrated here as involving rearrangement of only one chromosome. A more likely scenario is an unbalanced translocation, which is not shown in the figure. (D) Homogenously staining regions (HSR) and double minutes (DMs) are chromosomal structures that are generated as a consequence of gene amplification. HSRs are repeated units clustered at a single chromosomal locus (red), and DMs are unstable circular extrachromosomal DNA structures lacking a centromere or a telomere. In addition to these structures, amplicons can be present at a number of loci in the genome (not shown).

These genomic alterations have been termed structural variants, which are further classified as microscopic or submicroscopic, depending on the amount of DNA involved (5). The microscopic variations have historically been identified through chromosome banding techniques (6) and comprise at least 500 kb of DNA (7). Examples of these variants are whole-chromosome gain or loss (referred to as aneuploidy [7, 8]), translocation (change in location of a chromosomal segment [9]), deletion (deletion of a DNA segment relative to the rest of the chromosome [10]), duplication (a chromosomal segment occurs in two or more copies per haploid genome [11]), and inversion (reversal in orientation of a DNA segment compared to the rest of the chromosome [12, 13]). A schematic of structural variants resulting in copy number changes is shown in Fig. 1. With the development of more sophisticated tools, such as array-based comparative genomic hybridization (GGH) arrays (14–16), smaller variants (submicroscopic alterations) in the size range of 1 to 500 kb can be detected (5). Genome sequencing has further revealed small insertions and deletions (indels) spanning from 1 to 10,000 bp across the human genome which could cause considerable variability in the human population (17, 18).

The most common variant identified under submicroscopic alterations is copy number variation (CNV). CNV is defined as a genomic segment of more than 1 kb present at a variable copy number in comparison to a reference genome (19–22). The first studies documenting the genome-wide presence of CNVs in the normal human genome came from work in the laboratories of Lee (23) and Wigler (24). These studies described more than 200 large-scale CNVs (LCVs; about 100 kb or greater) in normal individuals. These studies also paved the way for the creation of the Database of Genomic Variants (DGV) in 2004, which catalogs all the human CNVs and structural variations present in healthy individuals.

The sequencing efforts from the International HapMap Consortium (25) and 1000 Genomes Project (26) have led to the identification and frequency determinations of novel CNVs in the human genome. CNVs are now known to contribute to 4.8% to 9.5% of the variability in the human genome (27, 28), which is more than what is accounted for by single nucleotide polymorphisms (SNPs; accounting for 0.1% of the variations) (29). Recently, the CNV map for the human genome was constructed (28), and it documented all the small- and large-scale CNVs present in normal healthy individuals. CNVs can either have no phenotypic consequences in individuals (4, 23, 24) or lead to adaptive benefits that have been observed in a wide range of species (5).

One of the major challenges in the field is to distinguish benign CNVs (events that do not lead to phenotypic consequences) from pathogenic CNVs that underlie diseases (30). Pathogenic CNVs are often associated with deleterious consequences because of an imbalance in gene dosage (31) and/or aberrant chromosomal structure (5, 7, 32, 33). Pathogenic CNVs have been associated with several disorders, including the following: obesity (34), diabetes (35), developmental disorders (36), psychiatric diseases (37) such as autism spectrum disorder (38), schizophrenia (39), and Alzheimer's disease (40, 41), and cancer (42–44). In this review, we focus mainly on copy number alterations observed in cancer and their functional implications.

CNVs can either be present in the germ line or can arise in phenotypically normal tissues and organs, which are referred to as somatic CNVs (45, 46). Instead of being randomly present in the genome, CNVs are preferentially found to occur in regions that are rich in low-copy-number repeats (segmental duplications) (47–50), heterochromatic areas (e.g., telomeres and centromeres), and replication origins and palindromic regions (28). There are several proposed mechanisms that underlie the generation of somatic CNVs: nonallelic homologous recombination (NAHR), nonhomologous end joining (NHEJ), defects in DNA replication, and DNA damage response and repair pathways. These mechanisms have been extensively discussed elsewhere; therefore, we refer our readers to several reviews (32, 33, 51).

In this review, we explore the relationship between copy number changes and biological consequences, with a particular focus on development and tissue homeostasis under physiological as well as pathological conditions. This review focuses on these relationships, especially in the context of cancer. We further discuss a recently discovered process driving transient site-specific copy number gains (TSSGs) in cancer cells and its implications during adaptive responses such as stress and chemotherapeutic sensitivity.

## COPY NUMBER CHANGES IN DEVELOPMENT AND PHYSIOLOGY

Chromosomal copy number changes and the associated gene amplifications and losses are observed during development in both lower and higher eukaryotes [reviewed in reference 7]. The appearance of CNVs during normal biology suggests that copy number changes can have important functional consequences. A common hypothesis is that increased gene dosages during development provide an advantage during selective pressures and environmental conditions (7). Here, we discuss examples from developmental biology and their relationships to functional impact. We also highlight the relationship between somatic CNVs and tissue homeostasis.

**FIG 2:**
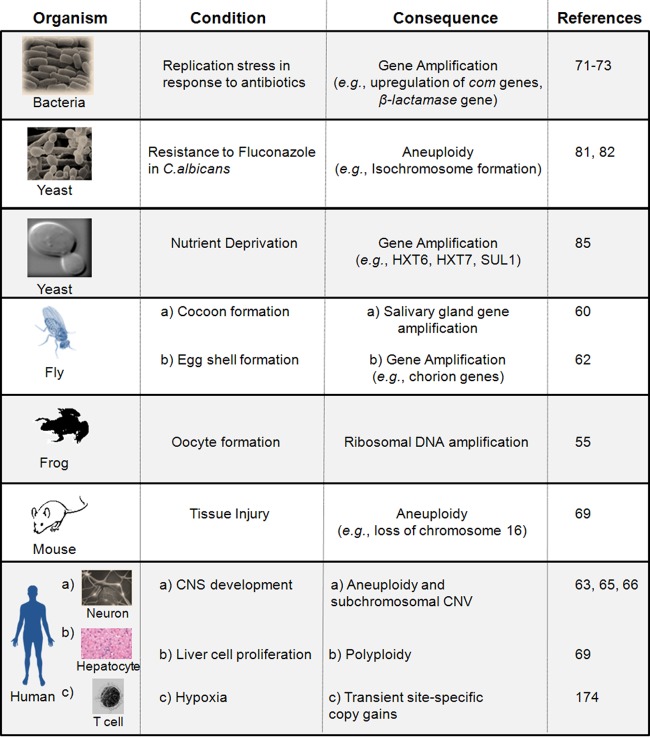
Copy number changes during normal development and physiological conditions. Representative copy number changes are shown for organisms and specific tissues under different developmental and physiological conditions. Please refer to text for detailed descriptions and corresponding references.

Several lower and higher eukaryotes use gene amplification to respond to cellular signals (Fig. 2). Electron microscopy studies in the early 1970s demonstrated that ribosomal genes are amplified for the production of large amounts of ribosomes required during early embryogenesis (52). Ribosomal DNA (rDNA) amplifications were observed during oocyte formation in amphibians such as Xenopus leavis (53–55), insects such as water beetles (56), molluscs (55), and in the macronuclear rDNA of Paramecium (57) and Tetrahymena (58). Thus, such an increase in rDNA synthesis to meet higher protein synthesis demands in different tissues highlights gene amplification as a common principle in developmental biology.

Besides rDNA, specific chromosomal regions identified as “DNA puffs” are amplified and expressed to form structural proteins required for cocoon formation in the salivary gland of sciarid flies (59, 60). Amplification of the DNA puffs occurs in response to the hormone ecdysone, which is required during larval development (60). Another example of gene amplification triggered by developmental signals can be observed during eggshell formation in Drosophila melanogaster (61). Eggshells require amplification of chorion genes in the follicle cells of the ovary, and these genes are expressed late in differentiation (61, 62). The amplifications of only specific chromosomal regions and genes and not the whole genome highlight the specific response that can occur across organisms. These examples suggest the ability of cellular cues to trigger these site-specific amplifications, which raises the question about what molecular mechanisms underpin this selective amplification across species.

Examples of copy number variations have been reported in various tissues in mammals. Using techniques such as spectral karyotyping (SKY), fluorescence *in situ* hybdridization (FISH), and single-cell sequencing approaches, various groups have reported both small- and large-scale changes in chromosomal copy numbers in mouse and human tissues, particularly in neurons, liver cells, and skin fibroblasts (Fig. 2). For example, approximately 33% of the neuroblasts in the embryonic mouse brain and 20% of neurons in the adult mouse cerebral cortex showed aneuploidy (63). The reduction in aneuploidy in the adult brain was hypothesized to be due to a neuroblast programmed cell death mechanism during brain development (64). Westra and colleagues also uncovered that 15 to 20% of neural progenitor cells in both mouse and human cerebella exhibited aneuploidy (65) (Fig. 2).

Additionally, high levels of subchromosomal CNVs (deletion and duplication events) were observed in the human frontal cortex neurons. Multiple copy number changes were noted within a small set of neurons, suggesting that CNVs might be restricted to either individual cells or specific neural lineages (66). These data suggest that the generation of copy number changes is an important process for achieving diversity in the neuronal populations during central nervous system development. However, this possibility has yet to be proven. It was reported that the transcripts arising from CNVs in the mouse brain are more tightly regulated than are other tissues such as lung, liver, heart, kidney, and testis (67). It would be important to determine the rate of correlation between CNVs and expression changes in the human brain and whether there are underlying functional consequences of the affected transcripts in generating neural diversity and plasticity.

Somatic CNVs are also observed in mammalian hepatocytes and skin. A study by Duncan and colleagues suggested that approximately 50% of normal adult hepatocytes have changes in chromosomal numbers (gains or losses) such that genetically diverse sets of cells are present in the liver (68, 69). However, single-cell next-generation sequencing has reported a lower level of aneuploidy (<5%) in cells of liver, skin, and human neurons (70). The differences in the reported levels of aneuploidies could reflect the different types of assays employed to follow copy number changes (i.e., FISH and SKY versus single-cell sequencing, respectively).

The genetic variation resulting from the changes in copy number could be a mechanism employed during tissue development in order to achieve diversity in cell populations. Copy number variations may allow developing tissues to adapt to cellular and growth requirements during tissue expansion and organ development. Another advantage for the observed CNVs could be to adapt to encountered metabolic or toxic challenges, especially by hepatocytes (see the discussion in “Mammals,” below). By identifying the regulatory features for regions undergoing CNV and the affected genes in different tissues, we would be able to understand tissue-specific gene expression and underlying diversity within tissues.

## COPY NUMBER CHANGES AS AN ADAPTIVE RESPONSE

Many studies in bacteria, yeast, and mammals have shown that copy number changes can arise as a consequence of selection, which may allow cells to exhibit an increased fitness and/or survival advantage. In this section, we discuss the relationship between different cellular conditions and the emergence of CNVs from different species (Fig. 2).

### Bacteria.

Acquisition of antibiotic resistance can occur through the uptake of foreign DNA harboring resistance genes through the bacterial competence pathway (71). A recent study by Slager et al. demonstrated that different species of bacteria could increase the copy number of genes involved in the competence pathway (*com* genes) in response to antibiotics causing replication stress (72). These genes are located closer to the origin of replication (OriC), and their amplification occurs through multiple origin firing events at the OriC, which increases their copy number and transcription rates. In Salmonella enterica serovar Typhimurium, gene amplification aids in the development of antibiotic resistance. Adaptation to the antibiotic cephalosporin occurred through amplification and increased gene dosage/expression of the β-lactamase gene (*bla*_TEM-1_ [73]). The enzyme β-lactamase results in the hydrolysis of cephalosporin (74, 75), which results in a reduced drug response.

These highlighted examples illustrate the impact selective pressure can have on DNA amplification and gene expression in bacteria (Fig. 2). Additional examples have been observed and are discussed in a review by Sandegren et al. (76). Taken together, the existing data illustrate the relationship between input signals and changes at distinct regions of the bacterial genome. In the future, it will be interesting to know if this selection is based on fitness or the result of targeted DNA replication in prokaryotes.

### Yeast.

Similar to bacteria, yeasts also exhibit changes in DNA content based on selective pressure. For example, gene rearrangements and copy number changes have been observed in Candida albicans when it is passaged through a murine host (77). It has been hypothesized that these changes in ploidy could generate the genetic and phenotypic diversity required for adaptation in the new host environment. Consistent with these observations, CNV has been associated with antifungal drug resistance and adaptive benefits (78, 79). For example, fluconazole treatment for C. albicans infection results in the development of whole-chromosome gains and aneuploidy (80). Upon CGH analyses for the copy number changes in 70 azole-resistant and -sensitive strains, Selmecki et al. found increased levels of aneuploidy in resistant strains (50%) compared to the sensitive ones (7.14%) (81, 82). Trisomies of chromosome 5, including a segmental aneuploidy consisting of an isochromosome (formed by the attachment of two left arms of chromosome 5 around a single centromere), were also associated with azole resistance. Gains of this isochromosome were associated with increased expression of genes involved in drug resistance (82). Some of these genes encoded efflux pump proteins involved in resistance: an ATP-binding cassette (ABC) transporter and a multidrug resistance transporter (83). Other genes were *ERG11* (a target of fluconazole [84]) and *TAC1* (a transcription factor that upregulates ABC gene expression [82]). There is a need to identify other structural variations and affected genes conferring a survival/adaptive advantage against antibiotics and whether these changes are conserved across other fungal species.

Consistent with gene amplification conferring a selective advantage, Saccharomyces cerevisiae cells exposed to nutrient deprivation exhibited gene amplifications that provided a cellular benefit (85). For example, glucose limitation in cultures resulted in the amplification of genes encoding glucose transporters (*HXT6* and *HXT7*), while sulfate limitation resulted in the amplification of *SUL1*, a gene that encodes a high-affinity sulfate transporter (Fig. 2). The question remains as to whether these physiological input signals are able to drive selective DNA gains through a hard-wired mechanism, as observed in mammalian cells (discussed in “TSSGs, Tumor Heterogeneity, and Cancer Evolution,” below), or are the result of random selection. Resolution of this issue could have a profound impact on our understanding of cellular fitness and responses to antibiotics.

### Mammals.

Mammals are no exception to selective pressures promoting copy number changes or copy number alterations that impact biological consequences. For example, the copy number of the human salivary amylase gene *AMY1*, which encodes an enzyme that aids in the hydrolysis of starch, is increased in populations that have a higher starch content in their diets compared to low-starch-consuming populations (86). The increased copy number of *AMY1* also correlated with increased salivary amylase protein levels. This illustrates how diet-induced selective pressures could influence copy number polymorphisms in mammals. Other examples and the role of copy number polymorphisms in human adaptation have been reviewed elsewhere (33, 87, 88). While these studies are correlative and suggest that the environment impacts selection, they have yet to be shown to be causal.

Increased or decreased copy numbers of certain genes can predispose an individual to diseases. For example, susceptibility of individuals to HIV/AIDS infection is increased in populations with a decreased copy number of the chemokine gene *CCL3L1*. This chemokine serves as a ligand for HIV coreceptor CCR5, which inhibits viral entry by binding to CCR5. However, HIV-resistant individuals show duplications of the CCL3L1 locus (17q21.1) and increased CCL3L1 copies imparting resistance to HIV infection (89). Other examples of CNVs promoting susceptibility to diseases can be found with psoriasis (associated with a copy number gain of the *β-defensin* gene [90, 91]), pancreatitis (a copy number gain of *PRSS1* [92]), and Crohn's disease (a copy number loss of *HBD-2* [93]), among others (20, 94). The question remains as to whether there are mechanisms that would allow such changes to occur immediately in response to stimuli in the population or whether they reflect some mutation that was selected over time.

Somatic mosaicism for CNVs within tissues can provide an adaptive response as well. CNVs within the liver can provide protection against tissue injury. Duncan et al. demonstrated in a chronic liver injury model that selective gene loss could provide resistance to liver injury (95). Deficiency of fumaryl acetoacetate hydrolase (encoded by *FAH*; the enzyme is required in tyrosine catabolism) causes a buildup of fatty acids and toxic metabolites that result in liver failure, known as tyrosinemia. Conversely, deletion of the genes encoding enzymes that function upstream of *FAH* (e.g., homogentisic acid dioxygenase [HGD]) is found to be protective for tyrosinemia. Mice deficient for *FAH* and heterozygous for a mutation in *HGD* can generate healthy normal hepatocytes. These injury-resistant, aneuploid hepatocytes (characterized by the loss of chromosome 16) are present in the liver and undergo expansion only when the liver is exposed to injury, demonstrating an adaptive response of cells to metabolic or toxic challenges.

Taken together, these few examples illustrate the CNVs present within populations and individual tissues and how these are associated with phenotypes. These data also emphasize the variations in the genome and how the environment and selective pressures can impact genetics. However, the question remains as to whether these genetic events occur after random selection or are the result of unidentified mechanisms that selectively alter the genetic landscape in response to external stimuli and, in turn, drive targeted *de novo* genetic changes.

## COPY NUMBER ALTERATIONS IN CANCER AND THEIR IMPLICATIONS IN ACQUIRED DRUG RESISTANCE

Copy number alterations involving whole chromosomes and/or specific chromosomal segments are frequently observed in cancer (96, 97). Gains/amplifications of oncogenes and loss/deletion of tumor suppressor genes have been historically found to be major drivers of tumor development. For example, amplifications of *EGFR* in gliomas (98), *MYCN* in neuroblastoma (99), *MYC* in acute myeloid leukemia (100), and *ERBB2* in breast (101), ovarian (102), and lung cancers (103) have been reported. Similarly, loss/deletions in tumor suppressor genes such as *PTEN* (104), *TP53* (105), and *VHL* (106) have been observed in a variety of tumors. The dependence of tumors on specific oncogenes for their proliferation and survival is referred to as oncogene addiction (107). By targeting these oncogenes, tumor cell growth becomes limiting or abrogated. For example, clinical success has been observed with the ERBB2 antibody trastuzumab (Herceptin) in the treatment of *ERBB2*-amplified breast cancer (108), crizotinib in the treatment of *MET*-amplified non-small cell lung cancer (109), and the epidermal growth factor receptor (EGFR) inhibitors gefitinib and erlotinib (which blocks the catalytic activity of EGFR) in lung cancer patients with *EGFR* mutations (110).

In addition to oncogene amplifications, copy number alterations of different chromosomal regions have been observed in cancer. A genome-wide analysis of copy number alterations in cancer demonstrated a total of 76,000 gains and 55,000 losses across the 3,131 cancer samples analyzed (96). A typical tumor type is comprised of 17% amplifications and 16% deletions, compared to less than 0.5% in normal samples (96). These data suggest that somatic copy number alterations are a frequent feature in cancer cells. Analyses across 17 tumor types demonstrated that 25% of the genome is affected by whole chromosome alterations and 10% of the genome by short chromosomal changes (focal events) in a typical tumor (96). Interestingly, the focally amplified regions often harbor known oncogenes (e.g., *MYC*, *CCND1*, *EGFR*, *NKX2-1*, and *KRAS*), while the focally deleted genomic loci contain tumor suppressor genes (*TP53*, *CDKN2A/B*, and *Rb1*). These observations suggest that the selective pressures associated with tumorigenesis might influence targeted amplification or deletion of specific regions within tumor cells instead of occurring randomly, which would be reminiscent of the observations seen in bacteria and yeasts (Fig. 2).

Focal amplifications can also harbor oncogenes or prosurvival genes that can influence drug responses. For example, ∼10% of cancers have a focal amplification of chromosome 1q21.2 that contains the antiapoptotic gene *MCL1* (96). Another focally amplified antiapoptotic gene that is observed in cancer is *BCL2L1* on chromosome 20q11.21 (96). Both of these genes are important for cell survival; hence, their amplification within tumors could confer a distinct survival advantage. Consistent with this notion, Beroukhim et al. demonstrated that increased expression of these genes protected tumor cells from chemotherapy (96).

Chromosomal alterations in several distinct regions also influence pathogenesis in different tumor types. For example, in multiple myeloma (MM), disease progression is characterized partly by the focal amplifications of a proximal region of chromosome 1q (chr 1q). Several studies have identified a region of 10 to 15 Mb that corresponds to a chr 1q12-23 amplicon in MM. This region contains a large number of genes with amplifications or deregulated expression involved in myeloma pathogenesis, including *CKS1B* (111, 112), *MUC1* (113), *MCL1* (114), *PDZK1* (115), *IL-6R* (116), *BCL9* (117), and *UBE2Q1* (118). The amplification of a drug-resistant oncogene, *CKS1B*, and the proximal chr 1q21 region has been reported in ∼40% of newly diagnosed MM cases and in 70% of patients with tumor relapse (119, 120). The gains observed in *CKS1B* are in the range of one to three copies (111, 112). These focal amplifications are associated with poor prognosis and reduced response to cisplatin therapy (111) (Table 1). Studies in cell cultures have further demonstrated that overexpression of *CKS1B* confers a reduced response to cancer chemotherapeutics (121). Similarly, amplification of the *PDZK1* gene within the chr 1q12-q22 region has been observed in primary cases of MM, and the overexpression of *PDZK1* in cells conferred resistance to melphalan-, vincristine-, and cisplatin-induced cell deaths (115) (Table 1).

**TABLE 1:** Partial list of amplified genes that impact drug resistance^*a*^

Cancer type	Therapeutic agent(s)	Gene(s) implicated in resistance (reference[s])
Multiple myeloma	Bortezomib, cisplatin	*CKS1B* (111, 121, 126)
	Melphalan, cisplatin, vincristine	*PDZK1* (115)
	Dexamethasone	*FGFR3* (127)
Ovarian	Cisplatin, CDK2 inhibitors	*CCNE1* (128, 142)
	Paclitaxel	*MDR1* (129, 130)
Lung	Gefitinib	*MET* (123, 125)
	Paclitaxel	*MDR1* (129, 130, 131)
	Crizotinib	*ALK*, *KIT* (132)
Breast	Trastuzumab	*MET* (133), *IQGAP1* (134)
Colorectal	Gefitinib	*MET* (124)
	5-Fluorouracil	*TMYS* (135)
CML	Imatinib	*BCR-ABL* (136)
Melanoma	Vemurafinib	*BRAF* (137, 138), *BCL2A1* (139)
Leukemia	Methotrexate	*DHFR* (140, 141)

a We apologize for not being able to cite or include all studies related to gene amplification and drug resistance.

Gene amplifications are associated with drug resistance in several tumors (122–141) (Table 1). For example, ovarian cancer patients with a chr 1q12-21 amplification are more resistant to cisplatin treatment (142, 143). Amplifications of cyclin E1 (CCNE1) are present in 25% of high-grade serous ovarian cancers and are associated with poor survival and impart resistance to CDK2 inhibitors (144) (Table 1). In the case of non-small cell lung cancer cells, an 11- to 13-fold-higher copy number of chr 7q21.12 was detected by CGH in an acquired paclitaxel-resistant lung cancer model (study NCI-H460/PTX250) compared with the parental cell line (study NCI-H460). Most of the genes within this region were also highly expressed, including a multidrug transporter gene, *MDR1/ABCB1* (131). These examples highlight how distinct regions in the genome are focally amplified and relate to altered patient outcome and cancer cell drug responses. Whether selective chromosomal alterations and gene amplifications in cells result from a stochastic process or occur in a directed manner in consequence to therapeutic pressure is yet to be determined.

## DNA AMPLIFICATION AND CANCER CHEMOTHERAPEUTIC RESISTANCE

**FIG 3:**
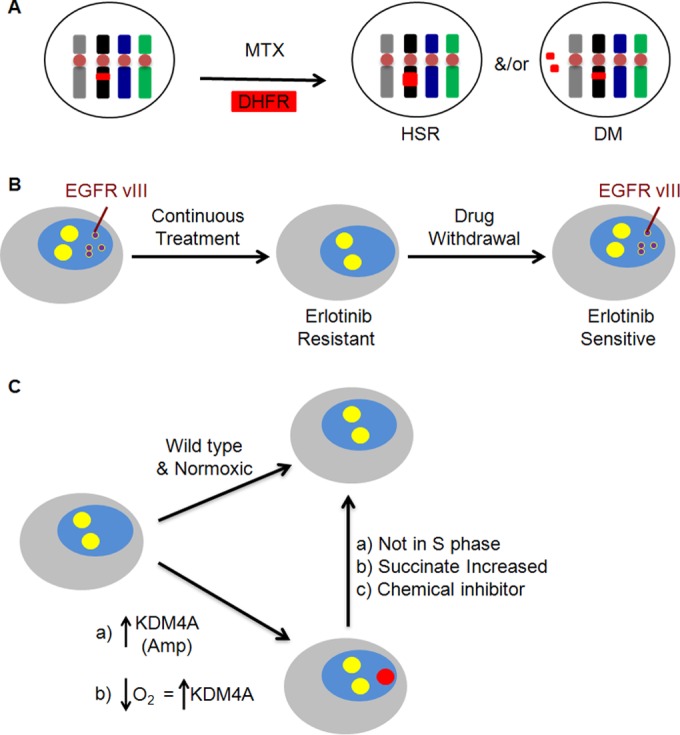
Permanent and transient adaptive changes under different cellular conditions. (A) Methotrexate treatment results in the amplification of the *DHFR* gene (shown in red). DHFR can persist either as a stable structure, such as an HSR, or as an unstable DM that is lost upon subsequent cell division. (B) Continued treatment of glioblastoma cells with a tyrosine kinase inhibitor such as erlotinib results in the loss of EGFR vIII-positive extrachromosomal DNA (red) and its reemergence upon drug removal. (C) Hypoxia or overexpression of histone demethylase KDM4A results in site-specific genome amplification (purple), which is generated every S phase. The amplification is reversible after KDM4 inhibitor treatment or with increased succinate dose. Studies related to these data are discussed in the text.

Gene amplification serves as a biochemical basis for drug resistance in mammalian cells. This relationship to resistance was first documented in seminal work by Hakala (145–147) and Fischer (148) in the 1950s. They isolated highly resistant tumor cells under the presence of increasing concentrations of the drug methotrexate (MTX). MTX competitively inhibits the enzyme dihydrofolate reductase (DHFR), which catalyzes the conversion of dihydrofolate to active tetrahydrofolate, which is required for the *de novo* synthesis of thymidine. They found that the drug-resistant cells had around 155 times the level of DHFR. They also found that the drug-resistant phenotype was unstable in murine sarcoma 180 cells, which coincided with the reduced DHFR enzymatic activity. Schimke's laboratory further characterized the mechanistic basis for the increased DHFR levels (149). It was shown that the cells developed resistance to MTX by overproduction of DHFR protein as a result of selective gene amplification (150). It was from the work of the Biedler and Spengler (151, 152) and Schimke (150, 153) laboratories in the 1970s that the presence of cytogenetic structures associated with MTX-resistant cells was demonstrated. They found that gene amplification accounts for the overproduction of DHFR in stable and unstable drug-resistant cells (Fig. 3A).

Gene amplification forms two common structures: extrachromosomal double minutes (DMs) and intrachromosomal homogenously staining regions (HSRs). DMs were first observed in lung cancer cells in 1962 (154). They are defined as chromatin bodies that lack centromeres and telomeres that are not transmitted to 100% of daughter cells during mitosis (155) (Fig. 1D). HSRs are chromosomal structures containing permanently integrated genes (Fig. 1D). These were first described by Biedler and Spengler in 1976 (152) in drug-resistant cells. DHFR was found to reside on HSRs in highly methotrexate-resistant CHO cells (156) and murine leukemia cells (157). Kauffmann et al. further showed that the amplified *DHFR* genes were associated with DMs in unstable MTX-resistant cells (158).

A large body of work has contributed to our understanding of the generation of DMs and HSRs (159–162). For example, Storlazzi et al. investigated the structures of *MYCN* amplifications by using eight neuroblastoma and two small cell carcinoma cell lines (162). The study provided evidence of generation of HSRs from DMs by an episome model wherein DNA segments were excised from a chromosome and then circularized and amplified to form DMs and chromosomally integrated to form HSRs. DMs are unstable and can be eliminated after drug treatment (163, 164); however, HSRs are more stable (165) (Fig. 1D and 3A). Amplified genes present on extrachromosomal DNA have been frequently observed in different tumor types (159, 166–168). The reversion of a malignant phenotype and cellular differentiation by the elimination of DMs has been shown extensively in a variety of tumors and cancer cell lines (167, 169, 170). Taken together, these observations demonstrate that transient gene amplifications can be an effective strategy for quick adaptation to selective pressures in tumor cells (Fig. 3A).

In a recent study by Nathanson et al., another example of drug-induced transient gene selection was demonstrated (Fig. 3B). In that study, oncogenes maintained on extrachromosomal DNA were transiently gained/lost in response to drug treatment (171). Glioblastoma patients harbor a constitutively active oncogenic variant of epidermal growth factor receptor (EGFR-vIII) that is formed by the in-frame deletion of exons 2 to 7 in the *EGFR* gene and found on extrachromosomal DNA (171, 172). The presence of EGFR-vIII makes tumor cells more sensitive to EGFR tyrosine kinase inhibitors (TKIs) (173). The continued treatment with EGFR TKIs (e.g., erlotinib) resulted in a loss of extrachromosomal EGFR-vIII, thus conferring resistance to the TKI. When the drug was withdrawn for a short period of time, there was an increase in EGFR-vIII on extrachromosomal DNA and, in turn, the cells were resensitized to erlotinib treatment (Fig. 3B). These data reiterate the reversibility of copy number gains and how transient copy number changes could impact chemotherapeutic response.

Furthermore, Nathanson and colleagues have suggested that instead of a continuous therapeutic regimen, a drug holiday during therapy might be a more effective mechanism to restore the sensitivity of tumor cells to drugs (171). These studies raise the possibility that chemotherapy could result in the selection of cells with gene amplifications, which allow them to survive under this drug-induced stress (Fig. 3). Therefore, understanding the mechanisms that result in transient or nonpermanent amplifications of *DHFR*, *EGFR*, and alike in cancer (Table 1) will have a profound impact on how we view copy number control as well as how we identify novel biomarkers and therapeutic targets for treating drug-resistant cancers.

## TSSGs, TUMOR HETEROGENEITY, AND CANCER EVOLUTION

There are frequent gains/amplifications observed across cancer genomes, which are often thought to be permanent events (33, 160). However, a recent discovery from our laboratory (174, 175) suggested a possible mechanism for the intratumoral heterogeneity of copy number alterations observed in tumors. This recent discovery could also provide a molecular basis for the emergence of amplified drug resistance genes and enhanced cancer cell survival.

Chromatin modulation plays an important role in replication fidelity (176, 177). A recent study demonstrated that alterations in chromatin states could modulate copy number gains at distinct regions in the genome (175). KDM4A/JMJD2A demethylates trimethylated histone H3 lysines 9 and 36 (H3K9/36me3) to a dimethylated state (K3K9/36me2) (178–182). KDM4A overexpression promoted faster S-phase progression and altered replication timing at specific regions in the genome in a catalytically dependent manner (175, 183). The regulation of S phase and replication timing were conserved from Caenorhabditis elegans to human cells and were the result of dysregulating specific HP1 members in the genome (HPL-2 in C. elegans and HP1γ in human cells) (183).

Even though the S phase was faster in mammalian cells, the rate of cell proliferation was the same, which was consistent with the observed slowing into the G_2_/M phase. This delayed G_2_/M was not associated with major genome instability. However, KDM4A overexpression directly generated site-specific copy gains of regions affiliated with drug resistance (e.g., chr 1q21-22) by altering methylation states and heterochromatin association. KDM4A was enriched at these sites and promoted their rereplication. Furthermore, direct H3K9/36me3 interference promoted site-specific copy number gain events. This study demonstrated for the first time that an enzyme has the ability to directly regulate copy number gain at specific regions in the genome and that the chromatin/methylation states play an essential role in the process (175) (Fig. 3C).

Since the copy number gain regions are not permanent and are only generated and present during S phase, they have been termed transient site-specific copy gains (174, 175, 184). Currently, we do not know the exact sizes of the rereplicated fragments and whether there are cellular checkpoints/machinery involved in their clearance. In fact, different cells in a population have differentially amplified regions, and certain regions are mutually exclusive. Furthermore, the rate that these fragments are removed as cells move through S phase is different (174). It is important to determine the molecular features (e.g., presence of repetitive elements, insulators, and other regulatory machinery) at and surrounding the rereplicated and regions that gain copies. These molecular details will help establish whether unique sequence features or chromatin states have a predilection for rereplication and whether site-specific copy gains can be integrated in the genome.

Stabilization of KDM4A as a result of exposure to cellular triggers such as hypoxia also resulted in TSSGs in cell lines, tumors, and normal primary cells (Fig. 2, T cells) (174). In fact, these copy number gains were found to be conserved at a syntenic region in zebrafish cells subjected to hypoxia. The return of cells to normoxia resulted in the reversion of copy number gains to the baseline levels (Fig. 3C). Hence, generation of transient copy number gains could be an adaptive cellular response of cells to external stresses or stimuli. These data provide a mechanism for heterogeneity within a cell population even though the same genetic event occurred in the population.

The stabilization of KDM4A upon hypoxic exposure or inhibition or loss of microRNAs regulating KDM4A promoted copy number gains of the drug resistance oncogene *CKS1B* (111, 112, 121, 185), which had a concomitant increase in transcripts (174, 191). When cells were returned to normoxic conditions, both copy number and transcripts of *CKS1B* returned to normal levels. Finally, we demonstrated that succinate (a natural inhibitor for the KDM4 class of demethylases [186]), chemical inhibition or microRNA-targeted depletion of KDM4A blocked the copy number gains upon hypoxic exposure (174, 191). These data emphasize the impact that metabolites could have on copy number gain, but most importantly, they identify a mechanism for blocking their generation (Fig. 3C). Since drug resistance oncogenes were increased, the inhibition of KDM4A may provide a novel mechanism for modulating TSSGs and provide a method for reducing 1q21 drug resistance-associated cancers.

The fact that transient exposure to elevated KDM4A can promote copy number gain that is only present during S phase suggests that other mechanisms must be present to remove the TSSGs. Similar mechanisms may be involved in the removal of extrachomosomal *DHFR* and *EGFR* amplifications. The TSSG data support the notion that chromosomal regions with specific genes that confer a survival advantage are amplified to protect the cell. Selectively amplifying genes that confer distinct advantages related either to cell survival, metabolism of drugs, mounting responses to counteract drug sensitivity, or features promoting tumorigenesis could aid in the evolution/adaptation of cancer cells. The question remains as to whether the classical oncogenes (e.g., *EGFR*, *MYC*, *ERBB2*, etc.) (Table 1) are subjected to site-specific copy gains in tumors and subsequent retention upon genetic, intrinsic, or extrinsic exposure. Some extrinsic cues could be therapeutic or metabolic challenge, stress conditions (such as hypoxia, nutrient deprivation), and vasculature and extracellular matrix plasticity. Future studies investigating their impact on TSSGs and gene amplification will be critical.

### Tumor heterogeneity.

Tumor heterogeneity presents a major diagnostic and therapeutic challenge in the treatment of cancer. Indeed, recent sequencing efforts with next-generation sequencing helped in the tracing of clonal lineages in tumors (187, 188). Focal gains or losses of chromosomes can result in diversity among cells in a tumor population (intratumoral heterogeneity [189]) as well as between tumors (intertumoral heterogeneity [189]). For example, next-generation sequencing of five bladder tumors from patients with transitional cell carcinoma of the urinary bladder showed genomic rearrangements and mutational heterogeneity within tumors (188). Whole-exome sequencing of samples from 18 patients with chronic lymphocytic leukemia (CLL) revealed the emergence of subclones within selected populations of cells treated with chemotherapy (190). These populations of cells might be more fit than their pretreatment counterparts and could contribute to relapse after therapy. Thus, identifying the mutational landscape before and after chemotherapy could not only identify mechanisms of tumor relapse but also help to design effective therapeutic options for the elimination of dominant subclones arising after chemotherapeutic selection pressures.

Another mechanism contributing to intratumoral heterogeneity could be the regulation of TSSGs from KDM4A levels, oxygen concentrations, cell division rates, metabolites, and KDM4A inhibition. Cells could be cycling at different rates in a tumor population, thereby affecting the rate at which rereplicated fragments are generated (Fig. 3C). Differential levels of KDM4A expression, hypoxia levels, or metabolic status in cells within a tumor population could also generate copy number gains at different rates, thereby affecting heterogeneity. We hypothesize that the site-specific rereplication events could be one of the characteristics acquired in specific population of cells during subclonal divergence. Specific environmental, metabolic, or therapeutic stress conditions can produce site-specific chromosomal alterations in the subclonal populations, which could either be transient, persisting only when the signal is there, or could eventually become integrated elsewhere in the genome upon subsequent genetic/epigenetic changes. TSSGs within specific cell populations could either influence the emergence of the dominant subclone or could go hand in hand with the germ line mutations occurring during tumor evolution. Whether these events result in the emergence of the fittest clone that promotes survival and if these sets of “fit” cells clonally expand after a therapeutic challenge is a hypothesis that needs to be investigated.

## CONCLUSIONS

CNVs influence the ability of normal cells to respond to physiological triggers and can serve as an adaptive strategy for a variety of responses, such as hypoxia, nutrient deprivation, toxic challenges, or cell survival and proliferation. Alterations in copy number often lead to diseases such as cancer, where the tumor cells can also coopt these aberrations as an adaptive response to amplify genes involved in chemotherapeutic resistance. It is important to determine whether the processes of generating copy number alterations under normal physiological, developmental, or pathological conditions are based on an active cell-directed and regulated mechanism or are the result of random aberrations that have occurred during cell division. Whether random or directed, it is important to understand that copy number changes are not always permanent. The recent discovery of a specific chromatin regulator controlling rereplication and site-specific copy number changes suggests that copy number changes can be regulated and are reversible. These transient site-specific copy gains may generate intratumoral heterogeneity that could have important consequences in chemotherapeutic sensitivity and patient outcome. Hence, identifying regulators of CNVs and delineating processes affected by CNVs will be important therapeutically.
